# Editorial: Advanced materials for the restoration and reconstruction of dental functions, volume II

**DOI:** 10.3389/fbioe.2023.1144275

**Published:** 2023-02-03

**Authors:** Bing Han, Chider Chen, Junchao Wei, Jianxun Ding, Tingting Yu

**Affiliations:** ^1^ Department of Orthodontics, School and Hospital of Stomatology, Peking University, Beijing, China; ^2^ Department of Oral and Maxillofacial Surgery and Pharmacology, School of Dental Medicine, University of Pennsylvania, Philadelphia, PA, United States; ^3^ School of Stomatology, Nanchang University, Nanchang, China; ^4^ Key Laboratory of Oral Biomedicine, Nanchang, China; ^5^ Key Laboratory of Polymer Ecomaterials, Changchun Institute of Applied Chemistry, Chinese Academy of Sciences, Changchun, China

**Keywords:** biomaterial, antibacterial material, regeneration, cartilage lubrication, drug delivery

There is a wide range of dental diseases, whether endodontic diseases, periodontal diseases, malocclusion, or oral and maxillofacial tumors, all of which cause psychological and physiological damage to patients to a certain extent. Thanks to the recent development in oral medicine, dentists have gained more methods and options for treating those diseases. This is not only due to the development of medical and theoretical techniques but also due to the development of materials science, which allows doctors to have more options to restore and reconstruct dental, periodontal, and maxillofacial functions, and it can be said that oral biomaterials have penetrated the clinical and scientific research in almost all dental specialties.

Currently, various types of polymers, organic, inorganic, and metallic materials, and other materials have been used in a wide range of clinical applications, including filling of dental defects, partial and complete dentures, oral implants, periodontal tissue regeneration, orthodontic appliances, surgical fracture fixation, and others. Further, with the development of dental materials science, researchers are not only satisfied with developing new types of materials but also fine-tuning and improving the specific materials according to characteristics of the disease itself to meet the clear clinical goals, such as enhancing antibacterial properties, bioactivity, osteoinductive, osteoconductive, and osteointegrative capacity, bonding properties, physical properties, and so forth. In addition, intelligent drug delivery systems are also gaining attention in clinical practice, and this helps us to target and quantitate drugs against diseases.

A total of 51 authors participated in preparing and applying advanced materials for functional reconstruction and restoration of the dental diseases in the Research Topic, which includes four reviews, three original articles, and one systematic review and network meta-analysis. (8,688 total visits; as of 2023/1/31). These studies include varieties of advanced materials, such as polymers, inorganic, organic, and metallic materials, and so on, for periodontal tissue regeneration, bone repair, drug delivery, and other specific clinical aims.

The articular cartilage carries different types and magnitudes of tensile and shear stress as the joints move thousands of times per day, and for this reason, the lubricity of cartilage determines whether it works effectively to protect the joint. An et al. reviewed the three mechanisms of cartilage lubrication called fluid film lubrication, boundary lubrication, and hydration lubrication. In all three models, hyaluronic acid, lubricin, and phospholipids play an essential synergistic role in cartilage lubrication, commonly found in the synovial fluid and cartilage of joints. Based on these principles, researchers combined hydrogels’ characteristics of strong water absorption, good biocompatibility, and other essential features and further obtained the hydrogels with good strength and lubricating ability by refining and improving them, such as double-network, nanocomposite, and ionic cross-linked hydrogels. In addition, hydrogels combined with stem cells, miRNA, and other components have the ability to promote cartilage regeneration. Besides, bio-lubricants showed excellent results in cartilage lubrication. Thus, the review summarizes the available solutions for joint diseases due to trauma and aging and provides a solid basis for subsequent research.

The incidence of joint diseases is increasing as the population ages. Total joint arthroplasty (TJA) is a surgical technique to remove part or all of a damaged joint and replace it with an artificial joint to relieve pain and restore function. It is an effective treatment strategy for end-stage joint disease. After the surgery, a severe prosthetic joint infection (PJI) commonly obtains due to microbial adhesion and biofilm formation, leading to a range of adverse events. For this reason, preventing, detecting, and treating artificial joint infections is paramount. The prevention of PJI can be achieved by combining biomaterials with antibacterial activity to improve bone cement, choosing prosthesis coating with an antibacterial effect, constructing anti-fouling coatings, and using bioactive glass and hydrogels as carriers to prevent bacterial adhesion and biofilm formation and kill bacteria. In addition, for the detection and diagnosis of PJI, researchers have developed hydrogel-based pH sensors and imaging systems of fluorescence-labeled polymeric nanoparticles to achieve non-invasive imaging, which is meaningful for the detection of PJI. Finally, for the treatment of PJI, the authors summarize the studies using hydrogels as carriers for a variety of antibacterial components, suggesting the importance of hydrogels as delivery systems. Besides, bone-targeted and bacterial-targeted therapies have also received certain attention (Xie et al.). In conclusion, this review summarizes the application of bioactive materials in PJI and demonstrates their advantages in prevention, detection and diagnosis, and intervention for clinical reference.

Periodontal disease is one of the most common oral diseases and is the leading cause of tooth loss in adults. So far, a comprehensive and systematic treatment strategy has been developed to treat periodontal disease, eliminating and controlling dental plaque as the core. Based on this, satisfactory and functional periodontal tissue regeneration has always been the goal of clinical treatment and scientific research, of which periodontal stem cells are the critical cells for periodontal regeneration, but due to long-term chronic inflammatory stimulation, the regenerative potential of periodontal tissue is often impaired. In addition, furcation involvement (FI) that occurs in molars is often severe and difficult to deal with, and the efficacy of conventional guided bone or tissue regeneration (GBR/GTR) is unstable. These unfavorable factors drove researchers to find targeted solutions. Wang et al. reduce the periodontal membrane-derived mesenchymal stem cell dysfunction caused by inflammation by combining low-intensity pulsed ultrasound (LIPUS), which helps to establish a suitable microenvironment for stem cells and promote periodontal bone formation and maturation of the bone matrix through anti-inflammation and pro-angiogenesis. In this article, the authors explored the degree of promoting periodontal regeneration of GTR combined with the non-invasive treatment of LIPUS in large animals like Beagle dogs, providing a reference for the clinical translation of LIPUS for the treatment of periodontal disease.

With the development of dental technology and the increasing demand for oral aesthetics and functionality, more and more patients are opting for dental implant restoration. Currently, the primary implant materials used in clinic are titanium (Ti) and Ti alloys, which are biocompatible and have good mechanical properties, but due to the biological surface inertia, Ti alloy itself does not have the ability to integrate with the bone or stimulate bone growth fully. The poor biocompatibility of Ti alloys is closely related to the naturally formed titanium oxide (TiO_2_) film being too thin. Therefore, surface treatment techniques are needed to modify the implant surface. Micro-arc oxidation (MAO) is a new electrochemical surface modification technology, which generates porous, rough, and firmly combined oxide films on metal surfaces. Not only that, through different electrolyte systems, but different kinds of oxide films can also be formed, further changing the characteristics of metal surface. The review summarized the ways in which MAO can be applied and their influencing parameters and introduced the properties of films prepared by different electrolyte systems (Wang et al.). In addition, considering that the MAO process is editable to some extent, the authors summarized some of the existing MAO combined with other therapeutic applications, which have a positive effect in improving the effectiveness and bioactivity of materials to a certain extent.

In addition to the surface oxide film, the structures of implants are also essential for their clinical application. Although Ti has good mechanical properties, the density of bone is not the same as Ti and has a porous structure, which exists a significant difference in elasticity modulus compared to metal, resulting in a stress shielding effect and thus affecting the restorative results. Therefore, researchers alter the pore size, shape, porosity, and pore structure of implants to increase the contact area for material−tissue interaction, regulate cell adhesion and migration, tissue vascularization, and nutrient and oxygen delivery to promote osteogenesis (Wang et al.). However, the manufacture of such refined materials remains a big challenge.

Three-dimensional (3D) printing, an additive manufacturing technology that allows computer-aided design and manufacturing with finely tuned overlapping layer by layer to give a 3D structure to meet application needs, has made modern dentistry increasingly digital and accessible. Tang et al. present the technical characteristics and respective advantages and disadvantages of the stereolithography apparatus (SLA), fused deposition modeling (FDM), selective laser sintering (SLS), selective laser melting (SLM), laminated object manufacturing (LOM), and digital laser processing (DLP) methods currently used in dentistry to provide a theoretical basis for clinical and scientific research. In addition, because 3D printing technology has the property of precision manufacturing, it satisfies clinical needs for aesthetics, restoring and improving function, and protecting vital structures, the study carried out the discussions based on this. In clinical practice, 3D printed models are more precise and easier to preserve and manage than traditional plaster models; in orthodontic treatment, 3D printed personalized lingual brackets are thinner and easier to fit and bond to the tooth surface, optimizing treatment outcomes; in addition, 3D printing enables the optimization of invisible appliances, the manufacture of surgical guides and the preparation of personalized, biocompatible implants. Although 3D printing deals with a variety of clinical problems, it is essential to note that there is still room for improvement in the accuracy of 3D printing technology due to factors, such as the step effect; also, the promotion and application of 3D printing technology is limited by the printing materials as the materials needed clinically are specific (Tang et al.). Although there are many problems, 3D technology still has a broad application prospect in dentistry.

To reduce the brittleness of zirconia (Zr) and the side effects of high hardness of Zr-containing materials on natural teeth, Sun et al. prepared two unique bioinspired nacre-like ceramic (yttria-stabilized Zr)−polymer (poly(methyl methacrylate)) composites and evaluated their cytotoxicity and bonding property. *In vitro* experiments showed that both materials have no cytotoxicity to human periodontal ligament fibroblasts (HPDLFs). The bonding strength of both composites was stronger than the control (Vita In-Ceram YZ), and the bonding strength increased with the polymer content. The excellent safety and bonding properties provide a basis for the clinical application of composites.

In addition, Chen et al. also focused on the bonding properties of dental materials and focused on cross-linkers, an essential substance that inactivates collagenase, enhancing the hardness of demineralized dentin, and keeping the structure of collagen fiber. They synthesized and evaluated the bonding performance with different applications of cross-linkers, different bonding systems, and different types of cross-linkers by systematic review and network meta-analysis. The results showed that the use of cross-linker enhanced the long-term bond strength and the bonding effect of etch-and-rinse (ER) system, with the bonding effect of dope-like compounds being better than other types of cross-linkers. The study has a guiding significance for the selection and application of cross-linkers in dentin bonding.

For the repair of bone defects, the use of therapeutic bio-tissue engineered scaffolds is a good solution, among which hydrogels are widely favored for their editability, biocompatibility, and easy accessibility, allowing researchers to synthesize and modify them towards a specific goal to enrich their function and make the treatment more targeted. By constructing a drug delivery system called alendronate (ALN)-conjoined injectable tetra-poly(ethylene glycol) (PEG) hydrogel, Chang et al. achieves sustained release of alendronate to promote stem cell growth, proliferation, and osteogenic differentiation, and the material has good biocompatibility. In conclusion, the study not only provides a new option for smart drug delivery systems but also offers a new option for the regenerative treatment of clinical bone defects.

All in all, this Research Topic covers not only the frontiers of antibacterial materials, cartilage lubrication materials, implants, and other medical functional repair and reconstruction materials, but also the fields of materials manufacturing, such as 3D printing technology, and drug delivery, which helps readers to understand the development of medical materials in a comprehensive and multi-faceted way. In addition, the above research is closely linked to materials science and clinical medicine, facilitating mutual learning and exchange between materials scientists and clinicians and promoting the clinical translation of theoretical research.

